# High intensity focused ultrasound treatment of small renal masses: Clinical effectiveness and technological advances

**DOI:** 10.4103/0970-1591.70561

**Published:** 2010

**Authors:** G. Nabi, C. Goodman, A. Melzer

**Affiliations:** 1 Department of Urology, University of Dundee, Scotland, DD1 9SY, UK; 2 Institute of Medical Science and Technology, University of Dundee, Scotland, DD1 9SY, UK

**Keywords:** Kidney, radiofrequency ablation, renal cancer, renal masses

## Abstract

The review summarises the technological advances in the application of high-intensity focused ultrasound for small renal masses presumed to be cancer including the systematic review of its clinical application. Current progress in the area of magnetic resonance image guided ultrasound ablation is also appraised. Specifically, organ tracking and real time monitoring of temperature changes during the treatment are discussed. Finally, areas of future research interest are outlined.

## INTRODUCTION

The advancements in new technologies for the management of renal cancer offer patients an opportunity for better and personalized care combined with shorter recovery time. Parallel progress in the fields of imaging science, biosensors, and computer technologies has made it possible to achieve an early diagnosis and improved point of care treatment for many cancers. The technological evolution, often driven by scientific and economic gains carry a potential to improve or replace traditional open surgical standards. A distinctive challenge in uro-oncology has been a rise in the detection of small asymptomatic renal masses due to cross-sectional imaging, specifically wide use of abdominal ultrasound. Most of these are malignant, however, a significant number are benign in nature and perhaps surgical removal is an over-treatment. Conversely, some of the small tumors may turn out to be aggressive in nature. Improvements in “converging technologies” of minimally access surgery and imaging have made it possible to ablate these lesions without surgical removal. High intensity focused ultrasound (HIFU) energy is an emerging minimally invasive technology in addition to existent ablative technique using radiofrequency wave or freezing (cryoablation) for small renal masses. The promise to be delivered extracorporeally is an attractive attribute of this technology. This may have a specific potential in elderly patients unfit for surgical intervention—a growing incidence of small renal masses diagnosed on routine imaging in this population.

The present review is aimed at:

1. Appraise the basic and advanced HIFU technology.
2. Systematically review clinical effectiveness.

## BASIC AND TECHNOLOGICAL ADVANCEMENTS IN HIFU TECHNOLOGY

### 

#### Mechanism of tissue ablation

HIFU is based on the principles of physical effect of ultrasound (US) energy on the tissue. A focused US beam causes mechanical vibrations in the tissues which produce heat. The thermal effect induces a rapid rise in the temperature within the focal volume of an US beam to cytotoxic levels by focal peak intensities from 5000 to 20,000 W cm^−2^, with operating frequencies of 0.8–3.2 MHz and thus selectively ablate a targeted tumor at net depth without any damage to the superficial tissues overlying the tumor or the surrounding parenchyma. The generated heat denatures the proteins and produces coagulative necrosis. The degree of necrosis depends on several factors: the applied power, the US frequency, transducer characteristics (shape, type, size, and number of probes), exposure time, spatial distribution of the field, absorption properties of the tissue, attenuation in the intervening tissue, acoustic reflection and refraction, and finally the perfusion rate in the targeted tissue.

The second effect of focused US energy is the production of cavitation-induced cellular damage (mechanical tissue lysis of cancer cells caused by the formation of microbubbles under high-tensile pressure). This mechanism can also be exploited for mediated gene transfer and drug delivery.[1,2] In addition, coagulative embolism of arteries or vein thrombosis causes ischemic damage in the immediate postcoagulative phase.

#### Delivery of energy

Extracorporeal systems: The energy, usually of >10,000 W cm^−2^ generated extracorporealy by either multiple or single piezoelectric elements is focused by lenses on the target lesion. There are two systems for extracorporeal HIFU used in experimental or clinical studies. The first one is from Storz Medical (Storz, Schaffhausen, Switzerland), where a parabolic reflector of 10-cm aperture focuses US energy generated by 1 MHz piezo up to a depth of 100 mm. The system has an integrated 3.5-MHz B-mode US transducer. The US beam is coupled into the body by a flexible polyurethane cushion filled with degassed water at 16 °C, which permits the variation of the skin-focal spot distance by altering its filling.[3] The second HIFU Chinese therapeutic system (Chonqing Haifu Co. Ltd., Chonqing, China) is composed of a patient table,, an operating console, and a treatment unit, situated under the table within a basin filled with degassed water to couple with US delivered to the patient. Patient is made to lie down over the water bath which has exchangeable ellipsoidal transducers of 12 or 15 cm diameter are installed around a central 3.5-MHz diagnostic transducer. This system offers therapeutic frequencies of 0.5, 1.2, and 1.5 MHz and permits varying focal lengths of 100–160 mm depending on the transducer in use. Wu *et al*. using an agreed treatment protocol and by exposing the targeted areas up to six times, could achieve an estimated site energy intensity of up to 20,000 W cm^−2^, enough to create cavitation and even bubble formation on real-time diagnostic imaging. The latter has been proposed as a marker of successful tissue ablation.[4]

Intracorporeal probes using laparoscopic approach: Few technological issues with the application of extracorporeal HIFU have generated interest in an alternate intracorporeal technique using laparoscopic or transcutaneous approach. Klingler *et al*. in a phase I study used laparoscopic approach through four 12-mm access ports. This was followed by the introduction of an 18-mm port (Ethicon, San Angelo, TX, USA) to introduce the laparoscopic HIFU system (Sonatherm, Misonix Inc., Fiarmigdale, NY, USA), which is composed of a treatment console, an articulated probe arm, a pump unit, and the laparoscopic probe.[5] HIFU energy is delivered by a truncated spherical shell 4-MHz transducer with a 30 × 13 mm^2^ aperture and a 35-mm focal length. Direct contact with the target provides a better assessment of the changes during the procedure and calibration, if required. In addition, the main advantage is that there are no acoustic interface between the probe and the tumor in contrast to extracorporeal HIFU.

#### Real-time monitoring

Tissue destruction depends on the level of temperature achievable at the target. Unpredictability of actual temperature changes at the focal point makes it essential to monitor the tissue destruction with real-time imaging during HIFU treatment. Moreover, this reduces the risk of any collateral damage and can be achieved by ultrasound (US) imaging or magnetic resonance imaging (MRI). During US monitoring, thermally induced gray-scale changes indicate temperature rise and hence tissue destruction, especially for peripheral tumors. Alternatively, MRI monitoring is used with images mapping temperature elevations. There are dedicated quantitative software programmes such as tissue change monitoring (TCM) system for Sonablate in prostate cancer treatment; however, use of this has not been described in the treatment of renal masses.

#### Future advancement

Robotic-assisted MRI-guided HFU system: Robotic technology and computational devices are used to achieve preplanning and navigation of surgical devices based on imaging data. Several advantages of robotic technology are: high accuracy, precision, repeatability, and alterations in desired trajectory. Magnetic resonance imaging technique used during intervention can acquire high-quality imaging data that can be used to precisely target the lesions; control the temperature in order to achieve the optimal tissue necrosis and tracking the position of the lesions. The addition of robotic assistance adds precision to the procedure.

MR-guided percutaneous interventions such as biopsies, drainage, and insertion of energetic probes for tumor ablation have been developed and clinically demonstrated with open bore, low field, and MR systems.[6] MRI-guided treatment of spine diseases was achieved with a 0.2-T open magnet.[7] In comparison, closed bore, high-field, MRI scanners ≥1 T have better spatial and temporal resolution, but patient access is more limited, hence, less feasible for interventions and demanding for robotics.

**Figure 1:**
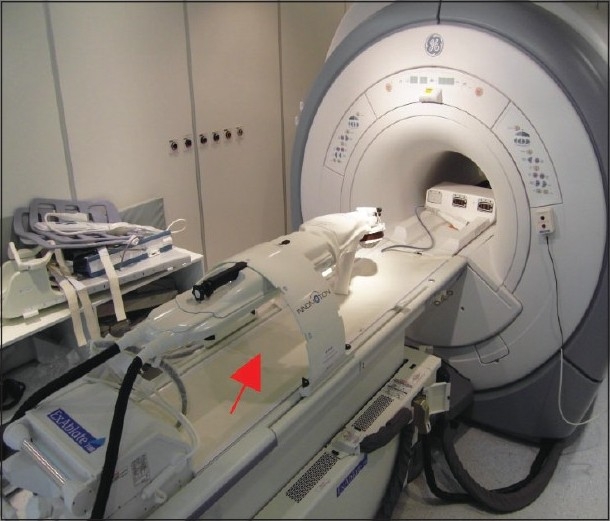
Robotic-assisted MRI-guided HIFU system (note robot-red arrow)

Various image compatible robotics have been developed such as fluoroscopy and computed tomography (CT) image-guided kidney biopsies.[8] Chinzei *et al*. have introduced a robotic assist system dedicated for the GE Signa SP “double donut” open MRI[9] and Gassert *et al*. reported on MRI compatible robotic for interaction with human arm motions.[10] The development of a fully MR-compatible robotic system started in 1998 at the Forschungszentrum Karlsruhe in collaboration with the University of Applied Sciences Gelsenkirchen, and in 2001, the German Cancer Research Center, Department of Radiation Physics (DKFZ, Heidelberg) joined the project in 2005. The final product development was performed by the start up company, Innomedic, Herxheim.[11,12] The MR robot INNOMOTION received CE mark in 2006 and is currently used for MRI-guided injections, biopsy, drainage, and tumor therapy. Our group is currently evaluated the feasibility of robotic-assisted MRI-guided focused US. The robotic system is capable of position of the FUS transducer exactly according to the MRI planning [Figure 1].

## SYSTEMATIC REVIEW OF CLINICAL EFFECTIVENESS OF HIFU TREATMENT

### 

#### Search methods of the reported literature

A sensitive search was developed with no language restriction. Studies reporting on HIFU both in the experimental and clinical settings were retrieved from following sources: PUBMED (1966–2009), EMBASE (1980–2009), and LILACS (1982–2009).

#### Outcomes

There were 52 reports in the English literature describing use of HIFU in kidney/renal masses. The breakdown of selected experimental (animal) and clinical (human) studies along with the countries of original studies are shown in Table 1. The popularity of HIFU as a minimally invasive approach in surgical oncology can be gauged by numerous recent reports in organs such as brain, breast, eye, prostate, bladder, uterus, liver, and so forth, showing no increase in cell dissemination.[6–9] This provides a glimpse of the uptake and dissemination of technology in the healthcare field. The clinical studies reporting on the effectiveness of HIFU used pathological criteria of achieving tissue necrosis as the end point of successful ablation.

**Table 1:** Selected studies reporting use of HIFU in the kidney tissues

Author (Reference)	Country	Animal (A) Human (H)	Outcomes	Extracorporeal (E) or Transcutaneous (T) or Laparoscopic (L)
Adams *et al*.[21]	USA	A	Pathological necrosis in 7/9 lesions	T
Hacker *et al*.[18]	Germany	H-Normal kidney tissue	Pathological changes in 43/43 kidneys	E
Illing *et al*.[25]	United Kingdom	Renal tumors (H)	Necrosis seen in 67%	E
Klinger *et al*.[5]	Austria	Renal tumors (H)	Complete necrosis in 9/10	L
Kohrmann *et al*.[33]	Germany	Renal tumors (H)	2/3 showed necrosis on MRI	E[6]
Orvieto *et al*.[34]	USA	A	All 16 lesions in 15 kidneys showed necrosis	L
Paterson *et al*.[35]	USA	A	All lesions showed necrosis	L
Roberts *et al*.[36]	USA	A (normal kidney tissue)	Lesions showed necrosis in all target lesions	T
Watkin *et al*.[37]	United Kingdom	A (normal kidneys)	Lesions seen in target lesions 40%	E
Wu *et al*.[23]	China	Advanced kidney tumors in 10 persons	Complete necrosis in three	E
Chapelon *et al*.[38]	France	A	Necrosis seen in 63%	E

Criteri a of pathological changes in the target tissue as an end point: Thermally induced histopathological changes remain the “gold standard” to assess the clinical effectiveness of ablative technologies in the target areas. HIFU energy causes progressive tissue changes in renal lesions. The immediate effects are intense congestion, hyperaemia, and alterations of the microcapillaries. The sub-cellular electron microscopy changes are: alterations of the mitochondria, ribosomes, and lysozymes. The process of necrosis sets in at day 2 and completes by day 7. At day 90, a complete fibrosis of the targeted area is observed.[10,11] Microcapillary damage in kidneys as seen microscopically is responsible for hemorrhage seen in patients treated for small renal masses with HIFU.[12] Histological analyses of tissue obtained from the excised small renal masses after HIFU application has confirmed “severe thermal tissue damage” defined as intravascular disruption of erythrocyte membranes, vacuolization of tumor and arterial smooth muscle cells, pycnosis and elongation of tumor cell nuclei, rupture of tumor cell membranes, and cell detachment, changes which correspond to complete tissue necrosis if the time elapsed from HIFU application and specimen removal is longer.[13] Irreversible heat damage has been corroborated by the negative NADH staining in snap-frozen tissue obtained before tissue fixation with formaldehyde after HIFU treatment.[5,13]

Outcomes of transcutaneous approach: Selected studies describing use of percutaneous aided by laparoscopic approach of HIFU application are shown in Table 1. The use of extracorporeal HIFU was reported in the treatment of a rabbit kidney.[14] Evidence of tissue necrosis in the form of well-demarcated coagulative necrosis, however, was seen in only in two of the nine tumors, when applied percutaneously in a rabbit model by others.[15] Similarly, Watkin *et al*. demonstrated tissue damage in 67% of the 18 treated pig kidneys.[16] In a different setting using canine model, HIFU application with 400 W power and 4-s pulse duration, and a calculated site intensity of 1430 W h^−1^ obtained coagulative necrosis of variable degree in the targeted area.[3] Recently, the use of microbubbles injected before percutaneous HIFU sonication of goat kidneys showed better necrosis rates than direct HIFU application.[17]

The results of the first human phase II study using the Storz system conducted by the University of Vienna were poor. Sixteen renal tumors treated with HIFU: 2 with curative intent and 14 prior to planned surgical resection. Histopathological necrosis as defined above of the specimens in terms of therapeutic effect was seen only in 9 out of 14 cases. All of these lesions had been exposed to the highest site intensities, and the histological damaged tissue only composed 15–35% of the targeted tissue.[5] In a similar clinical study, Hacker *et al*. treated 19 patients with renal cell carcinoma before surgical removal, focusing HIFU to healthy renal tissue as well. Thermal damage of the removed specimen was variable and poor; seen just in 15 out of 19 specimens. Moreover, they could not correlate the energy administered and lesion size suggesting difficulties in achieving the desired energy sufficient enough to cause necrosis in the target lesions.[10]

In an another phase II, two trial using the Chongqing system, Wu *et al*. reported significant symptomatic improvement (decrease in pain and cessation of hematuria) in palliative treatment of 13 advanced renal cell carcinoma applying percutaneous HIFU. They admitted that treatment was considered inadequate in 10 patients.[17] Similar discouraging results were reported from UK in eight patients treated using the same system. Only four out of six kidneys showed radiological evidence of treatment effect on MRI 12 days after HIFU application and just one out of four removed kidneys showed histological confirmed ablation.[18]

Outcomes of laparoscopic approach: The selected outcomes of laparoscopic approach are shown in Table 1. Klinger *et al*. in a clinical phase I study, using laparoscopic HIFU approach treated 10 patients with solitary renal masses. Two of the renal lesions were 9 cm in size. In this case, HIFU was applied just as a marker lesion before radical laparoscopic nephrectomy. The remaining renal masses were of a median size of 2.2 cm and were treated with a “curative intent” applying HIFU to the entire tumor with a margin of 2–3 mm of surrounding normal parenchyma. Laparoscopic partial nephrectomy was performed in seven of these tumors and one was left *in situ* in a patient with high comorbidities. The median HIFU treatment time was 19 min (range 8–42) in small renal masses. In the first two patients, close to an area of probe placement, just a 2–3 mm of vital tissue was seen on histopathology with coagulative necrosis in rest of the tumors. The authors explained this phenomenon to an excessive cooling of the probe during the procedure. They changed the treatment protocol for the rest of the treatment group on the basis of this observation. Complete necrosis was seen in the four remaining removed cases (57%). The nonexcised tumours were followed up by CT scans at 6-month intervals. The core biopsies of these lesions showed coagulative necrosis. There were no enhancement on follow-up imaging and shrinking of the lesion was observed.[5]

Complications: There have been no serious side effects in the treatment of renal cancer using HIFU;[3] just two patients had grade III skin lesions[20] after transcutaneous HIFU, but the most common type of skin toxicity is less than 1 cm blister or track at the treatment site.[21] Changes in laboratory tests are also found to be nonsignificant.[17]

## DISCUSSION

There have been significant encouraging technological improvements of HIFU treatment to manage small renal masses. Two prototype systems such as Storz and Chonqing Haifu have made it possible to conduct phase 1 and 2 studies in animal and patients. Although initial results are far from desired, these studies have shown the safety in targeting the renal tumors. Moreover, it is possible now to focus renal lesions using both extracorporeal and laparoscopic approaches as confirmed by histological evidence.[12]

Extracoporeal application of HIFU remains a challenge due to several factors. A combination of patient-related and technological limitations interfere with the power emitted by the US probe and the energy arriving to the targeted area. The focal length, type, and characteristics of the tissue to be crossed through by the energy waves, variable vascularization of the kidney and its mobility as well as the limitation of proximity of air (gut) or bone (ribs)[22] are some of the technical and anatomical challenges to be overcome for HIFU treatment of small renal masses to become a part of clinical management.

The second challenge of percutaneous HIFU application, possibly more important from clinical effectiveness point of view, in the absence of a relaible radiological method of monitoring the effect of HIFU in real time. The focus of ongoing research is to find more fixed devices coupled with respiratory movements trying to save absorption of US energy from nontargeted tissues such as ribs, fat, or muscles; MRI with its advantage of providing information within seconds of energy application, is being more extensively proposed as a guide to the treatment compared with regular US.[23] Real-time organ tracking is currently under development and the new MRI-guided focused ultrasound (MRgFUS) system by InSightec (Haifa, Israel) carries potential to compensate the respiratory motions. Phillips has introduced a new HIFU system integrated in the MRI patient table featuring HIFU beam steering.

Mobility has been partly corrected using multichannel focused US systems, trying to combine motion tracking and feedback electronic steering of the HIFU beam and multiprobe systems of small-aperture confocal HIFU transducers that also theoretically permit more flexible targeting.[10]

The poor clinical outcomes, in particular inconsistent histopathological results, could be explained by the combination of drawbacks of the currently available HIFU technologies. A robust technological research guided by vigorous clinical evaluation is still needed before HIFU could be considered suitable for the clinical treatment of renal cancer. Till then this remains to be used in experimental settings.

Laparoscopic-guided application of HIFU facilitates resolutions of many of the problems facing the extracorporeal approach. Using such an approach, Klinger *et al*. reported a successful outcome of tissue necrosis just for peripheral tumors not larger than 3.5 cm in size.[5] The method avoids clamping of hilar vessesl and punctures of renal lesion as necessary for other ablative techniques and certainly opens a window to clinical research. This technique needs further evaluation in a randomized controled trial setting comparing this with other ablative methods and active surveillance.

Postablation follow-up of Small Renal Masses SRM using radiology remains to be defined. Patients are generally followed-up by contrast-enhanced CT and MRI. The other methods such as PET and microbubble contrast-enhanced US are under evaluation.[24] In addition, microbubbles increase the ablation efficiency and the visibility of tissue destruction.[25] A general lack of consensus regarding the best way to follow-up these lesions after treatment as with other ablative and non-ablative techniques exists,[26] and the role of follow-up biopsy in contrast-enhanced area at the site of ablation needs further evaluation.[24]

**Figure 2:**
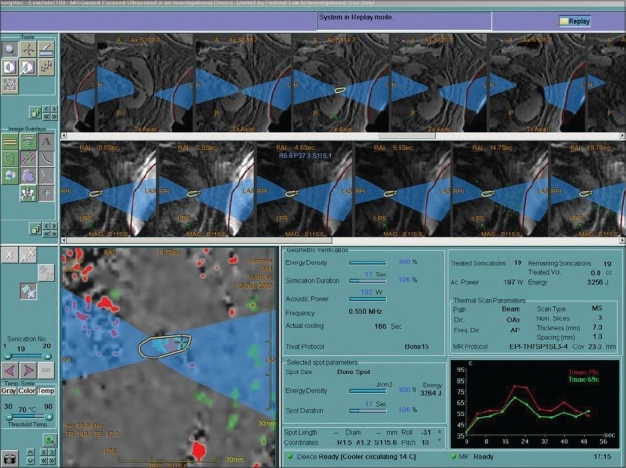
MR-guided focusing of kidney. Note monitoring of temperature changes (red arrow)

Among the various advantages of HIFU applications; repeated use due to lack of cumulative dose effect remains the main attraction; however, need of general anesthesia, especially for laparoscopic approach limits this strategy. MRI-guided focused ultrasound would be optimal as it provides real-time temperature mapping alongside precise tumor delineation for target definition [Figure 2] and accurate three-dimensional treatment planning.[28] MRgFUS is approved in Europe, Japan, and USA for the treatment of uterine fibroids and is currently undergoing clinical trials for the treatment of breast, liver, prostate, and brain cancer and for the palliation of pain in bone metastasis.[29]

Focused US induces temporary change of vascular and cell membrane permeability and mechanically triggers the release process of drug delivery systems, i.e., liposomes[30] and polymers.[31] The aim of current research is the combination of MRgFUS tumor ablation under accurate targeting of the thermal ablation process through mapping of the achieved temperatures, and triggered drug release for an increased effective drug concentration in the treatment zone but lower systemic concentration and reduced side effects.

The challenges and focus of future HIFU research for small renal masses are:

- Standardize the pulse and power levels which ascertain tissue death of malignant cells.
- Number of probes needed for transcuataneous or laparoscopic approach to cause optimal effect.
- Tracking movements of organs such as kidney in extracorporeal application
- Optimal time and extent of necrosis achievable without causing any unnecessary destruction to the normal tissue.
- Long-term follow-up to evaluate complications, recurrences free survival, quality of life, and cost effectiveness for the healthcare organization and society.
- Multicenter randomized controlled trails with the established techniques (open or laparoscopic partial nephrectomy) considered as gold standard,[28] evolving technology (cryotherapy and radiofrequency,[29] and active surveillance, an option with many proponents to manage small renal masses.

## CONCLUSIONS

HIFU is a fast improving technology with potential to establish itself as the most minimally invasive among the currently available techniques. Poor preliminary clinical results can be explained by the limitation of the technology which is an area of focused research. MRgFUS provides nonaccess thermal ablation with continuous temperature mapping and once fast organ tracking becomes available it has the potential to become an alternative to surgical resection of malignant renal tumors.
